# Exploiting gene × gene interaction in linkage analysis

**DOI:** 10.1186/1753-6561-1-s1-s64

**Published:** 2007-12-18

**Authors:** Yungui Huang, Christopher W Bartlett, Alberto M Segre, Jeffrey R O'Connell, LaVonne Mangin, Veronica J Vieland

**Affiliations:** 1 Center for Quantitative and Computational Biology, Columbus Children's Research Institute, 700 Children's Drive, Columbus, Ohio 43205, USA; 2 Department of Pediatrics, College of Medicine, The Ohio State University, 700 Children's Drive, Columbus, Ohio 43205, USA; 3 Department of Computer Science, College of Liberal Arts, University of Iowa, 14 MacLean Hall, Iowa City, Iowa 52242, USA; 4 Department of Medicine, University of Maryland, University of Maryland Medical Center, N3W42, 22 South Greene Street, Baltimore, Maryland 21201, USA

## Abstract

When two genes interact to cause a clinically important phenotype, it would seem reasonable to expect that we could leverage genotypic information at one of the loci in order to improve our ability to detect the other. We were therefore interested in extending the posterior probability of linkage (PPL), a class of linkage statistics we have been developing over the past decade, in order to explicitly allow for gene × gene interaction. In this report we utilize a new implementation of the PPL incorporating liability classes (LCs), which provide a direct parameterization of gene × gene interaction by allowing the penetrances at the locus being evaluated to depend upon measured genotypes at a known locus. With knowledge of the generating model for the simulated rheumatoid arthritis (RA) data, we selected two loci for examination: Locus A, which in interaction with the HLA-DR antigen locus affects risk of the dichotomous RA phenotype; and Locus E, which in interaction with DR affects quantitative levels of the anti-CCP phenotype. The data comprised nuclear families of two parents and an affected sib pair (ASP). Our results confirm theoretical work suggesting that gene × gene interactions CANNOT be leveraged to improve linkage detection for dichotomous traits based on affecteds-only data structures. However, incorporation of DR-based LCs did lead to appreciably higher quantitative trait PPLs. This suggests that gene × gene interactions could be effectively used in quantitative trait analyses even when families have been ascertained as ASPs for a related dichotomous trait.

## Background

There is considerable interest in modeling gene × gene interaction for purposes of mapping and understanding complex traits (e.g., [1-3]). However, Vieland and Huang [4] showed that for two-locus (2L) models and data restricted to individuals who are affected (hereafter referred to as affecteds-only data) (e.g., ASPs), gene × gene interactions do not result in distinctive patterns in identity-by-descent (IBD) sharing, and therefore linkage analysis using ASPs cannot be used to distinguish interaction from independent gene effects (heterogeneity), with a few exceptions unlikely to be relevant to complex disorders. They suggested as a corollary that methods designed to exploit known interactions for purposes of mapping new genes would not be fruitful in ASPs. On the other hand, if the generating model involves more than two loci, then gene × gene interactions may predict specific structure in the (marginal) 2L IBD matrix even in affecteds-only data [2-5]. However, even in this case whether numerical effects would be substantial enough to provide meaningful benefits has not been systematically investigated.

In this paper, we ask whether it is possible to improve on our ability to map a new trait gene via linkage analysis by using the causal variants at a known risk locus, under conditions of gene × gene interaction, using a newly implemented extension of the posterior probability of linkage (PPL) to measure the strength of evidence for (or against) linkage.

## Methods

### Family data

Analyses were performed on only the first 500 families from the first 50 replicates to reduce PPL computation time because using the full set of data and number of replications was wasteful of resources for our purposes here (At the time of the initial draft of the paper, it would take up to a couple of weeks to complete one replicate. Since then, the program has improved greatly and reduced the time to less than a day [6].). Based on inspection of the answer file, we selected marker STRP16_6 for dichotomous trait (rheumatoid arthritis, RA) linkage analysis. This marker is at 27.44 cM on chromosome 16, 1.15 cM away from Locus A. Because Locus A and DR interact to increase RA risk (see the "Risk Multipliers" table in the answer file), we then used the genotypes at DR to classify individuals into liability classes. For computational convenience [6], we restricted attention to just two liability classes: LC1 comprised individuals with two DR4 alleles (the high risk group); LC2 comprised the remaining individuals (a low risk group).

For quantitative trait (QT) analysis, the phenotype anti-cyclic citrullinated peptide antibody (anti-CCP) was chosen, and Locus E was evaluated for linkage. Again, the generating model included an interactive effect of DR and Locus E on anti-CCP levels. Anti-CCP measures were standardized on the basis of all available parental values; no other changes to the phenotypes were made. Linkage analysis was applied to marker STRP18_22, located at 92.9 cM on chromosome 18, which is 1.4 cM away from Locus E. As above, any individual with two copies of the high-risk DR4 allele was coded as being in LC1, all other individuals were coded to be in LC2.

### Statistical analysis

The PPL is on the probability scale, can readily incorporate prior information, and is particularly suited to the accumulation of evidence across multiple, potentially heterogeneous, data sets [7-9]. The unknown trait model is treated as a vector of nuisance parameters, and integrated out of the constituent likelihoods [10,11]; thus the method is essentially model-free, while retaining the strengths of likelihood-based analysis. Further, in application to quantitative traits, this framework does not assume normality at the population level or require population parameter estimates [12,13] in order to address ascertainment.

As described in detail elsewhere [10,11], the PPL can be computed from an ordinary LOD score, with the unknown parameters of the trait model integrated out rather than fixed at arbitrary values. The PPL can therefore in principle be extended to incorporate any form of likelihood for which LODs can be calculated. Thusfar we have extended the original dichotomous trait PPL [7,14], which already allowed for locus heterogeneity under the admixture model [15], to include allowance for linkage disequilibrium [16], sex-specific recombination [17,18], quantitative traits [13], combined quantitative/dichotomous traits (within the same pedigree) [12], implemented in both two-point and and/or multipoint forms [19].

The standard dichotomous trait PPL is parameterized in terms of the (sex-averaged) recombination fraction, the admixture parameter, a disease allele frequency, and three penetrances (one for each genotype, assuming a two-allele locus). The standard quantitative trait (QT) PPL is parameterized similarly, except that instead of three penetrances, the likelihood is written as a function of three genotypic means and three genotypic variances [12,13]. (In the present application we have set the three variances equal to one another.) As elsewhere, we assume a 2% prior probability of linkage [20]. Thus PPLs > 2% represent (some level of) evidence in favor of linkage; while PPLs < 2% represent (some level of) evidence against linkage. The PPL is on the probability scale, and is therefore bounded by [0, 1]. For comparison purposes, we also report MODs [21], which are LODs parameterized identically to the PPLs, then maximized over all parameters in the model (whereas the PPL is integrated over these same parameters).

Here we extend the PPL once again, to allow different penetrances for individuals in different LCs. The new extension of the PPL allows covariate-dependent penetrances. Specifically, we assign individuals to liability classes (LCs) based on covariate status. In the present application, we use this parameterization to condition on the causal genotype at the known risk locus DR; however, the same model could be used to condition on other covariates, such as age or sex [22]. We then include a separate penetrance vector in the likelihood for each LC in the model. These penetrance vectors are then integrated over, rather than fixed, to obtain a marginal posterior probability. In the dichotomous trait analyses, we have constrained the A-locus penetrances for individuals in LC1 to be greater than or equal to the corresponding penetrances in LC2, for each A-locus genotype, respectively. In the QT analyses, we have constrained the genotypic means for individuals in LC1 to be greater than or equal to means for individuals LC2, again, for each E-locus genotype, respectively. We have recently implemented a suite of PPL statistics in a new package, KELVIN, designed for distributed parallel computation over the parameter space [6,23]. KELVIN is based on a re-engineered version of VITESSE [24,25], thusfar incorporating two-point and multipoint linkage analysis of dichotomous and/or quantitative traits, marker-trait linkage disequilibrium, and LCs. Exportable software is currently under development. Unsupported and platform specific version can be made available by contacting the corresponding author.

## Results

Linkage analyses at Locus A ignoring genetic information at DR (i.e., without LCs) yielded an average PPL of 2.04% (SD ≈ 0.0195), or essentially no evidence for or against linkage. By comparison, the average PPL utilizing LCs based on DR genotype is 2.28% (SD ≈ 0.0418), which is numerically higher though virtually the same in practical terms, and still yields essentially no evidence for linkage (see Table 1). The within-replicate average PPL difference is 0.25% (SD ≈ 0.0231), although only 7 out of 50 replicates have higher LC-PPLs than PPLs. There is a slight (though not statistically significant) tendency for the LC-PPL to actually be lower in each replicate when LCs are used. By contrast, The MOD roughly doubles (Table 1) in magnitude because it is maximized over extra parameters, but it still gives results that would not be interpreted as evidence for linkage.

**Table 1:** Average PPLs with and without DR liability classes (LCs)

	PPL (SD)^a^		MOD (SD)	
		
	No LCs	With LCs	No LCs	With LCs
Locus A (Dichotomous Trait)	0.020 (0.020)	0.023 (0.042)	0.43 (0.46)	0.89 (0.64)
Locus E (Quantitative Trait)	0.24 (0.29)	0.44 (0.36)	2.87 (1.32)	4.81 (1.71)

^a^By convention, PPLs of 3% or less are reported to three decimal places; while PPLs > 3% are reported to two.

In stark contrast with the dichotomous trait results, the quantitative trait linkage analyses at Locus E shows marked increases in the average evidence for linkage with the addition of DR information from 24% (SD ≈ 0.29) to 44% (SD ≈ 0.36) (see Table 1). The MODs are also larger when DR information is incorporated. In order to ensure the noticeably higher LC-PPL is not inflation simply due to the increased number of parameters in the model, we applied the same statistical analysis to the unlinked data obtained from the first markers on each chromosome 1–5 and 7; these markers were not annotated as being linked to any of the simulated phenotypes. The average QT-PPL was less than the 2% prior probability of linkage (data not shown), indicating evidence against linkage. This illustrates that the observed increase in the PPL at the original "linked" marker is not an artifact of including the additional penetrance parameters in the model. By contrast, the MODs increase when the additional parameters of the LCs are maximized over, at the unlinked markers as well as at the linked marker. This illustrates a key distinction in handling of nuisance parameters by integration (as with the PPL) versus maximization (as with the MOD).

## Conclusion

Gene × gene interaction (or other covariate dependencies) can be directly represented in standard linkage likelihoods using LCs [22]. While theoretical considerations suggest that modeling gene × gene interactions in affecteds-only data is moot for dichotomous traits under two-locus models, in the context of quantitative trait analysis as well as under models with more than two loci, the situation could be fundamentally different. By extending the PPL to incorporate LCs for both dichotomous and quantitative trait analyses, we have shown that, under these generating conditions, utilizing genotypic information at the DR locus when evaluating the evidence for or against linkage to Locus A has no impact on our ability to detect Locus A using dichotomous trait analyses; whereas, incorporating information on DR is beneficial in detecting Locus E using quantitative trait analysis.

## Discussion

By extending the PPL to include a direct representation of gene × gene interaction (or other covariate dependencies), we have shown that under the generating conditions used in this simulation, incorporation of measured genotypes at a known locus does not improve our ability to detect linkage to another interacting locus for a dichotomous trait. These results are fully consistent with previous theoretical work on affecteds-only data under two-locus models, despite the fact that the generating model involves more than two loci and does include some unaffected individuals (among the parents). It appears that, nevertheless, there is virtually no information in nuclear families, that include only affected offspring, regarding the genetic architecture of the trait; and that as a result, utilizing information at one locus when evaluating a second is largely moot. This does not represent a specific limitation of the PPL, but rather, a limitation of the data structures provided in this simulation. See Kotti et al. [26] and Larkin et al. [27] for similar conclusions based on a variety of other statistical approaches.

We do have evidence that incorporation of gene × gene interactions in this manner may be more helpful in larger pedigrees, including even nuclear families with unaffected as well as affected offspring (data not shown). However, even in larger pedigrees, the utility of measured genotypes in forming LCs will be governed in part by the relative representation of different classes of individuals within the data set (the distribution of phenotypes, genotype at the marker being evaluated, and genotypes at the "risk" locus being conditioned on); and this in turn is a function of the underlying architecture of the trait together with the sampling frame and ascertainment criteria. Further research is needed to investigate other generating models that may potentially benefit from the use of LCs to model gene × gene interactions.

By contrast, the simulated data clearly support the use of LCs to model gene × gene interactions in quantitative trait linkage, even when the data are ascertained as ASPs for a related dichotomous trait. Apparently in this case sufficient variation in the phenotype still remains so that allowing genotypic means to depend on genotypes at a known risk locus can lead to stronger evidence for linkage at a second, interacting locus.

## List of Abbreviations

Anti-CCP – anti-cyclic citrullinated peptide antibody

ASP – affected sibling pair

DR – the HLA-DR antigen locus

LC – liability class

MOD – a LOD score maximized over all trait parameters

PPL – posterior probability of linkage

RA – rheumatoid arthritis

## Competing interests

The author(s) declare that they have no competing interests.

## Authors' contributions

YH performed all programming tasks for Kelvin, based on Vitesse code provided by JRO; YH also had substantial influence on design and interpretation and wrote large sections of the manuscript. CWB and VJV provided statistical methods and played a major role in writing/revising the manuscript. AMS and JRO collaborated on code development and numerical methods of the statistics; LM developed and applied specific informatics support; VJV supervised all aspects of the project. All authors have read and approved the final manuscript.
